# Treatment of Mucous Polypus of the Velum Palati

**Published:** 1875-04

**Authors:** 


### ARTICLE VI.

### *Treatment of Mucous Polypus of the Velum Palati.*

A case is recorded (*Bull Gen. de Therap.*,) of this affection, by Dr. Meplain, of Moulins, in which a young man, 30 years of age, suffered from a small tumour of bright red colour, soft consistence, not pulsating, slightly pedunculated situated at the junction of the hard and soft palate, a little to the left of the median line. Its duration had been about three weeks, and it had grown rather rapidly from the size of a lentil, when it was first noticed by the patient, to that of a bean, when Dr. Meplain first saw it. It interfered with deglutition and speech, and was liable to frequent hemorrhages after food had been taken. No cause could be assigned for its development. M. Meplain determined to treat it with repeated application of caustic, and for this purpose selected chromic acid, which he painted over its surface four days running. No improvement resulting, he next attempted to remove it with a pair of scissors curved on the flat; the hemorrhage was moderate, and was easily arrested with a compress moistened with perchloride of iron. In three weeks the patient returned with the tumour, which had reached its original size. On this occasion he seized it with a pair of dressing forceps, and endeavoured to tear it away. Moderate bleeding only followed, and this was soon stopped with a little acid gargle. Eight days after the patient again presented himself, and M. Meplain, somewhat discouraged by his failures, recommended him to try frequent application of carbolic acid. This was found to be very difficult, troublesome, and painful but it appeared to check the growth of the tumor. Once more it was removed with the scissors, and once more it returned in the course of a week. Galvano-caustic he had not the means of applying, but he proposed the actual cautery, to which the patient strongly objected. He therefore at length determined to inject with acetic acid. He only threw in one drop with an Anel's syringe, but the effect was most satisfactory. The pain was acute for a short time, but the tumour gradually wasted away and did not return.—*Practitioner.*

